# Usnic acid brief exposure suppresses cariogenic properties and complexity of *Streptococcus mutans* biofilms

**DOI:** 10.1016/j.bioflm.2024.100241

**Published:** 2024-11-30

**Authors:** Santosh Pandit, Mi-A Kim, Ji-Eun Jung, Hyeon-Mi Choi, Jae-Gyu Jeon

**Affiliations:** a Systems and Synthetic Biology Division, Department of Life Sciences, Chalmers University of Technology, SE-412 96, Gothenburg, Sweden; b Department of Preventive Dentistry, School of Dentistry, Jeonbuk National University, Jeonju, Republic of Korea; c Department of Dentistry, Presbyterian Medical Center, Jeonju, Republic of Korea

**Keywords:** *Streptococcus mutans*, Biofilms, Dental caries, Usnic acid

## Abstract

Bacterial biofilms are highly structured surface associated architecture of micro-colonies, which are strongly bonded with the exopolymeric matrix of their own synthesis. These exopolymeric substances, mainly exopolysaccharides (EPS) initially assist the bacterial adhesion and finally form a bridge over the microcolonies to protect them from environmental assaults and antimicrobial exposure. Bacterial cells in dental biofilm metabolize dietary carbohydrates and produce organic acids. The blanket of exopolysaccharides over the bacterial communities hinders the buffering by saliva, contributing to the initiation of tooth decay followed by the progression of dental caries. Considering the current interest towards the use of natural antimicrobial agents to disarm the cariogenic properties of dental biofilm, this study evaluated the antimicrobial activity and the effect of twice daily brief exposure (1 min) of usnic acid on acid production, acid tolerance and development of 3-dimensional architecture of *Streptococcus mutans* biofilm. Herein, biofilms were briefly treated twice daily during biofilm development and biofilms were analyzed by using biochemical, microbiological and microscopic examination. Results obtained in this study showed a significant reduction in virulence properties of biofilm cells treated with usnic acid in compared to non-treated biofilms. Furthermore, twice daily brief exposure of usnic acid significantly disrupted the acid production and reduced the complexity of *Streptococcus mutans* biofilm by disrupting the EPS production. Brief exposure of usnic acid inhibited the production of glucosyltransferase (GTF) enzymes and their enzymatic activity leading to inhibition in production of EPS on the biofilm matrix. In conclusion, usnic acid treatment reduced the cariogenic properties and complexity of *S. mutans* biofilm by inhibiting acid production, acid tolerance and disrupting extracellular polysaccharide (EPS) formation, indicating its potential for preventing dental caries.

## 1 Introduction

Biofilms are highly hydrated structures formed by the communities of bacteria [1,2]. Bacterial cells in the biofilm matrix are encased with extracellular polymeric substances by their own synthesis [3]. The EPS matrix is mainly composed of polysaccharides, protein, and eDNA [4,5]. Bacterial cells in the biofilm are covered by exopolymeric substances, which protects them from environmental assaults and antimicrobial exposure [6]. Among the composition of EPS matrix, exo-polysaccharides are believed to be a major component in biofilm matrix [7]. The adhesive nature of exo-polysaccharide provides the binding site for the bacterial colonization and facilitates in the development of complex multispecies biofilm matrix [8,9].

*Streptococcus mutans* is considered as an etiological cariogenic bacterium on dental biofilms [10,11]. Bacterial cells in dental biofilm metabolize dietary carbohydrates and produce large amounts of organic acids via glycolysis [12]. The acidic environment promotes the selective growth of highly aciduric bacteria resulting in an ecological shift to establish highly cariogenic biofilm [13]. The high acidic environment in the oral cavity starts to demineralize the tooth enamel and ultimately leads to dentinal caries. The glucosyltransferase enzyme produced by *S. mutans* synthesizes polysaccharides by catalyzing the dietary sucrose. The produced polysaccharide helps to gather both inter and intra species bacterial strains on the biofilm resulting in formation of complex micro colonies [14]. Furthermore, these polysaccharides serve as a nutrient for the biofilm bacteria promoting metabolic activities within the biofilm matrix [15]. Hence, the proper strategy to mitigate the dental biofilm matrix is considered as an effective way to prevent dental caries.

Usnic acid, a secondary lichen metabolite, holds a strong antimicrobial property specially against gram positive bacteria [[16], [17], [18]]. Usnic acid has been widely studied considering its potential to be used in medicinal products, oral hygiene products and skin creams to control various infections [16,19]. Previous studies have demonstrated the strong antimicrobial efficacy of usnic acid against the various bacterial strains including oral pathogens [[16], [17], [18], [19], [20]]. However, most of these studies are tested against the planktonic state of bacteria [17,20]. Despite the potential of usnic acid to be used in oral care products such as mouth wash and toothpaste, its effect against the cariogenic biofilms is rarely investigated. Hence, the aim of this study is to evaluate the effect of twice daily topical application of usnic acid on cariogenic properties and development of complex architecture of *Streptococcus mutans* biofilm.

## 2 Materials and methods

### 2.1 Bacterial strains and media

A total of 9 bacterial strains were used in this study. This includes, *Streptococcus mutans* UA 159, *Streptococcus oralis* ATCC 5037, *Streptococcus mutans* KCTC 3298, *Streptococcus mutans* KCTC 3300, *Streptococcus sobrinus* KCTC 3308, *Streptococcus sobrinus* KCTC 3288, *Streptococcus cricetus* KCTC 3292, *Streptococcus rattus* KCTC 3294 and *Streptococcus sanguis* KCTC 3284. All strains were used to determine minimum inhibitory concentration and minimum bactericidal concentration. Only *Streptococcus mutans* UA159 was used as a model organism to evaluate the anti-biofilm and anti-cariogenic activity of usnic acid. *S. mutans* UA159 (ATCC 700610; serotype c) was grown in tryptone-yeast extract broth (TYE broth; 2.5 % tryptone and 1.5 % yeast extract, pH 7.0) supplemented with 1 % glucose (for planktonic cells) or 1 % sucrose (for biofilms). All chemicals and culture media used in this study including usnic acid was purchased from Sigma Aldrich (USA). Usnic acid was dissolved in dimethyl sulfoxide (DMSO) and used as final 4 % DMSO as a working vehicle concentration (control).

### 2.2 Determination of minimum inhibitory concentration (MIC)

Minimum inhibitory concentration was determined as described previously [21]. Briefly, inoculum suspension was prepared from overnight growth of bacterial culture. The bacterial inoculum was diluted with fresh culture medium and used 1-1.5 × 10^6^ CFU/ml. The final concentration of usnic acid ranged from 0.48 to 250 μg/ml was prepared with a series of two-fold dilution. MIC was defined as a lowest concentration that restricts the bacterial growth to an absorbance lower than 0.05 at OD 550 nm. To determine the MBC, 100 μl of aliquot from all the well above than MIC were dropped into agar plates. The MBC was defined as the lowest concentration that did not permit any visible growth on the agar plate after 48 h of incubation.

### 2.3 Biofilm formation and brief exposure with usnic acid

*S. mutans* biofilms were formed on hydroxyapatite (HA) discs (2.93 cm^2^; Clarkson Chromatography Products, South Williamsport, PA, USA) as described previously [22]. Briefly, HA discs were transferred into 24-well plates containing 1 % sucrose (w/v) ultrafiltered (10 kDa molecular weight cut-off) tryptone yeast-extract (UTE) broth (pH 7.0) with *S. mutans* UA159 (2-5 × 10^6^ CFU/ml). The biofilms were grown at 37 °C with 5 % CO_2_. After 22 h of biofilm growth, the culture medium was changed twice daily until it was 74 h. These untreated biofilms were used to evaluate the effect of usnic acid against cariogenic properties. To determine the effect of brief exposure, HA discs treated with various concentrations of usnic acid (0, 50, 100, 150 and 200 μg/ml) for 1 min were transferred into 24 well containing 2.8 ml of 1 % sucrose UTE broth with *S. mutans* inoculum (2-5 × 10^6^ CFU/ml). After the 22 h of biofilm growth, biofilm was treated briefly with vehicle control (4 % DMSO), 50, 100, 150 and 200 μg/ml of usnic acid followed by the media change. The treatment and medium replacement were carried out twice a day (1 min each, a total of 6 times) until the age of 74 h.

### 2.4 Effect of usnic acid on acid production, acid tolerance and EPS production by *S. mutans* biofilm

To determine the anti-acidogenic effect of usnic acid, the glycolytic pH drop assay was carried out. Briefly, 74 h old untreated biofilm formed on HA discs were transferred into 12 ml of salt solution (pH 7.0) containing vehicle control (4 % DMSO), 50, 100, 150 and 200 μg/ml (final concentration in 12 ml) of usnic acid. The pH of the solution was adjusted to 7.2 by adding 0.2 M KOH. Glucose (final concentration: 1 % (w/v)) was added to start the glycolysis metabolism and drop in pH was monitored over 120 min. Anti-acidogenic effect of usnic acid was evaluated by rate of hydrogen ion production by biofilm and its accumulation over the experimental period. The rate of hydrogen ion production and final accumulation were calculated by using pH values measured until first 40 min (0–40 min) and 120 min respectively on the pH drop curves.

To examine the anti-aciduric effect of usnic acid, its effect on F-ATPase enzymatic activity was evaluated of biofilm bacteria. Briefly, 74 h of biofilms were collected and homogenized by sonication. Homogenized biofilm cells were permeabilized by 10 % toluene (v/v) followed by two freezing and thawing cycles. F-ATPase activity was measured in terms of the release of phosphate in the following reaction mixture: 75 mM of Tris-maleate buffer (pH 7.0) containing 5 mM adenosine triphosphate (ATP), 10 mM magnesium chloride, permeabilized biofilm cells, and different concentration of usnic acid. The amount of phosphate released over the 30 min reaction time was determined as described previously [23].

The activity of crude GTF to form water soluble and insoluble polysaccharides in the presence of sucrose was examined to evaluate the effect of usnic acid on GTF activity. Briefly, the reaction mixture (1 ml) consisted of the following: (a) 300 μl of the enzyme, (b) 175 μl of 0.01 M potassium phosphate buffer (pH 6.8), (c) 500 μl of 0.01 M potassium phosphate buffer (pH 6.8) containing 5 % sucrose, and (d) various concentration of usnic acid (0, 50, 100, 150 and 200 μg/ml) were incubated for 24 h. Water soluble and insoluble polysaccharides were extracted by water and 1 N sodium hydroxide and precipitated by ethanol. Water soluble and insoluble polysaccharides were determined by phenol sulphuric acid assay as described previously [24].

### 2.5 Effect of usnic acid brief exposure on acid production and composition of *S. mutans* biofilm

To determine the effect of brief usnic acid exposure, biofilm was formed on HA discs and treated twice daily (9 a.m. and 6 p.m.; from day 1) until the 74-h age. Biofilm were exposed to the various concentration of usnic acid for 1 min followed by two dip washed with autoclave water to remove the excess agent and transferred into the fresh medium. The pH of old culture medium was examined to determine the anti-acidogenic effect of brief usnic acid exposure. To analyze biofilms, twice daily treated biofilms were removed and homogenized by sonication. The homogenized biofilm suspension was diluted serially and plated into agar plates to count CFUs. The homogenized biofilm suspension was also used to determine the dry weight, water insoluble polysaccharides and iodophilic polysaccharides. Briefly, water insoluble polysaccharides were extracted by using 1 N sodium hydroxide, precipitated by using ethanol and quantitated by using phenol-sulphuric acid method. Iodophilic polysaccharides were extracted and quantitated by the glycogen assay as described previously [22].

### 2.6 Scanning electron microscopy analysis

Twice daily treated biofilms were dip-washed with 0.1 M cacodylate buffer and fixed with 2.5 % glutaraldehyde for 30 min and followed by the post fixation with 1 % osmium tetroxide for 1 h. The fixed biofilms were dehydrated with graded series of ethanol (30–100 %) and infiltered with nitrogen gas immediately before the sputter coating with gold-palladium. The coated biofilm sample were examined under the scanning electron microscope (5000X).

### 2.7 Confocal laser scanning microscopy analysis

Confocal laser microscopy was used to examine the effect of usnic acid brief exposure on *S. mutans* biofilm matrix as described previously [12,25]. Briefly, 1 μM of labeled Alexa fluor® 647-labeled dextran conjugate was added to the culture medium during the biofilm formation and maturation to stain exopolysaccharides. After 74 h these biofilms were exposed to 2.5 μM SYTO® 9 green-fluorescent nucleic acid stain. Four independent experiments were performed and 20 image stacks (512 × 512 pixel) from 5 sites per experiment were collected (n = 20). Biofilms (bacterial bio-volume and thickness, and EPS bio-volume and thickness) were quantified from the confocal stacks by using COMSTAT [26]. The three-dimensional architecture of the biofilms was visualized using Imaris software (Bitplane, Zurich, Switzerland).

### 2.8 Effect of usnic acid brief exposure on GTF production

Supernatant of homogenized suspension of biofilms were used for the quantification of GTF in biofilm. Collected supernatants were concentrated under the vacuum and the amount of GTF B, C and D were quantified by using ELISA as described previously [27]. Briefly, 96-well flat-bottom polystyrene microtiter plate (Nunc, Roskilde, Denmark) was coated with 100 μl of coating buffer (0.05 M sodium carbonate-bicarbonate buffer with pH 9.6) and 100 μl of supernatant. The plate was incubated at 4 °C overnight and then blocked with 5 % skim milk in PBS for 30 min at room temperature (RT). After washing 3 times with PBS, 200 ng of monoclonal antibodies against GTF B, C and D that had been previously prepared in 100 μl of 5 % skim milk was added and incubated for 1 h at 37 °C, followed by 3 washes with PBS. Then, a secondary alkaline phosphatase-labeled goat anti-mouse immunoglobulin G antibody (Sigma Chemical) was added, and the wells were washed 4 times with PBS. Color was developed with an alkaline phosphatase substrate and the wells were read at 405 nm using an ELISA reader (Packard Instrument, Downers Grove, IL). The amount of GTFs was measured and determined by absorbance value of OD 405 nm.

### 2.9 Statistical analysis

At least three individual experiments were performed for each assay and data presented as mean ± standard deviation. The intergroup differences were estimated by one-way ANOVA, followed by a post hoc multiple comparison (Tukey test). Values were considered statistically significant at *P* < 0.05.

## 3 Results

### 3.1 Antimicrobial effect of usnic acid

MIC and MBC value of usnic acid against the various oral bacteria is presented on Table 1. Usnic acid showed strong antimicrobial activity against all the tested oral bacterial strains. MIC value of usnic acid against *S. mutans*, *S. sobrinus*, *S. cricetus* and *S. rattus* was range from 1.95 to 3.9 μg/ml. Usnic acid showed strong antimicrobial activity against the *S. oralis* and *S. sanguis* showing the MIC value 0.95 μg/ml. MBC value of usnic acid against *S. mutans*, *S. cricetus* and *S. rattus* are higher than 250 μg/ml while 3.9 μg/ml for *S. sobrinus* and 1.95 μg/ml for *S. oralis* and *S. sanguis*.

**Table 1:** MIC and MBC of usnic acid against the various oral pathogen.

Bacterial strain	MIC (μg/ml)	MBC (μg/ml)
*Streptococcus oralis* ATCC 5037	0.98	1.95
*Streptococcus mutans* UA 159	1.95	>250
*Streptococcus mutans* KCTC 3298	1.95	>250
*Streptococcus mutans* KCTC 3300	3.9	>250
*Streptococcus sobrinus* KCTC 3308	3.9	3.9
*Streptococcus sobrinus* KCTC 3288	3.9	3.9
*S. cricetus* KCTC 3292	1.95	>250
*S. rattus* KCTC 3294	1.95	>250
*Streptococcus sanguis* KCTC 3284	0.98	1.95

### 3.2 Anti-virulece properties of usnic acid

**Fig. 1:**
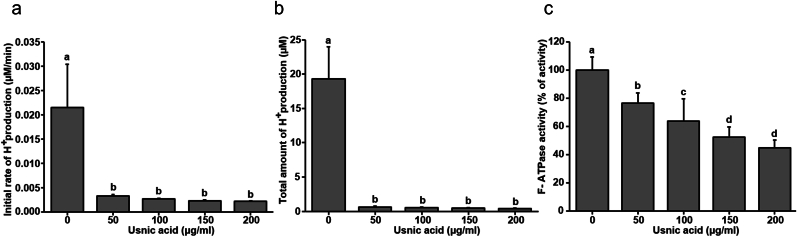
Effect of usnic acid on acidogenicity and aciduricity of *S. mutans* biofilm cells. (a) Inhibitory effect of usnic acid on initial rate of H^+^ ion production by mature biofilm cells. (b) Influence of usnic acid on total amount of H^+^ ion concentration by mature biofilms cells in the 2 h of time period. (c) Effect of usnic acid on F-ATPase activity of mature *S. mutans* biofilm cells. Data represent mean ± standard deviation of three individual experiments. Values followed by the same superscripts are not significantly different from each other.

**Fig. 2:**
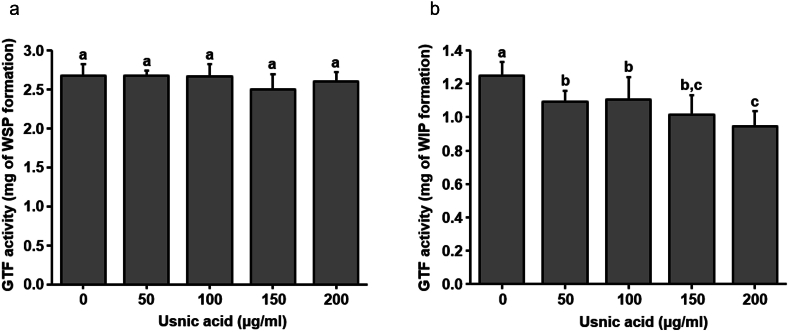
Influence of usnic acid on activity of crude glucosyltransferase enzyme of *S. mutans*. (a) Effect of usnic acid on water soluble polysaccharides formation by glucosyltransferase enzyme. (b) Effect of usnic acid on water insoluble polysaccharides formation by glucosyltransferase enzyme. Data represent mean ± standard deviation of three individual experiments. Values followed by the same superscripts are not significantly different from each other.

Usnic acid showed a strong anti-acidogenic effect against *S. mutans* biofilm. As shown in Fig. 1a and b, the entire concentration of usnic acid used in this study inhibited both initial rate of acid production and accumulation of H^+^ ions by biofilm cells. Furthermore, usnic acid also inhibited the F-ATPase activity of biofilm cells in concentration dependent manner. As shown in Fig. 1C, usnic acid showed 25–55 % of inhibition on ATPase activity of *S. mutans* biofilm cells. Where, 25 % of inhibition was shown by 50 μg/ml and 55 % of inhibition showed by 200 μg/ml of usnic acid. Furthermore, the effect of usnic acid on GTF enzymatic activity was tested to examine its potential to prevent glucan production. As shown in Fig. 2a, usnic acid showed no inhibitory effect on the production of water-soluble polysaccharides. In contrast, all the tested concentration of usnic acid affected the production of water insoluble polysaccharides (Fig. 2b).

### 3.3 Effect of brief exposure of usnic acid on *S. mutans* biofilm

**Fig. 3:**
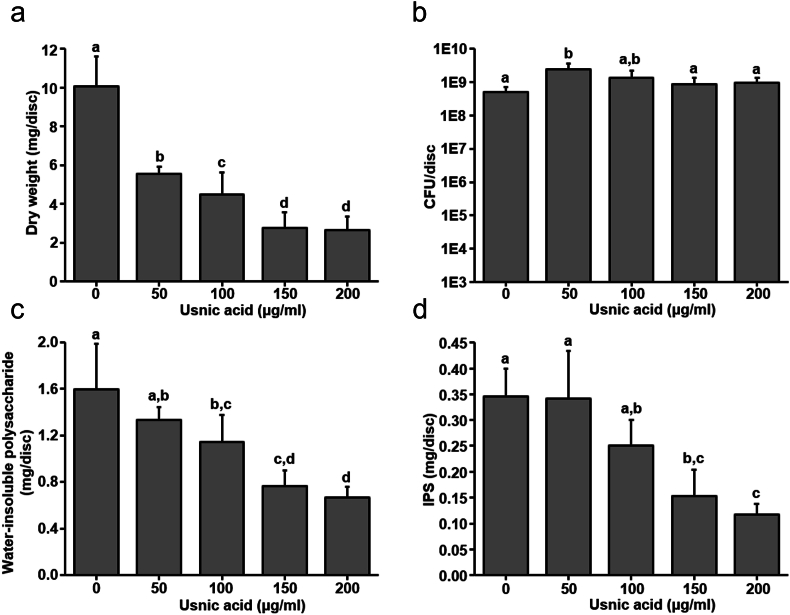
Influence of twice daily exposure of usnic acid on biochemical and bacterial composition of mature biofilm. (a) Effect of twice daily brief exposure of total biomass of biofilm. (b) Influence of twice daily brief exposure on viability of biofilms bacterial cells. Inhibitory effect of twice daily brief exposure of usnic acid on (c) Water insoluble polysaccharides and (d) iodophilic polysaccharides production by biofilm cells during the mature biofilm development. Data represent mean ± standard deviation of three individual experiments. Values followed by the same superscripts are not significantly different from each other.

**Fig. 4:**
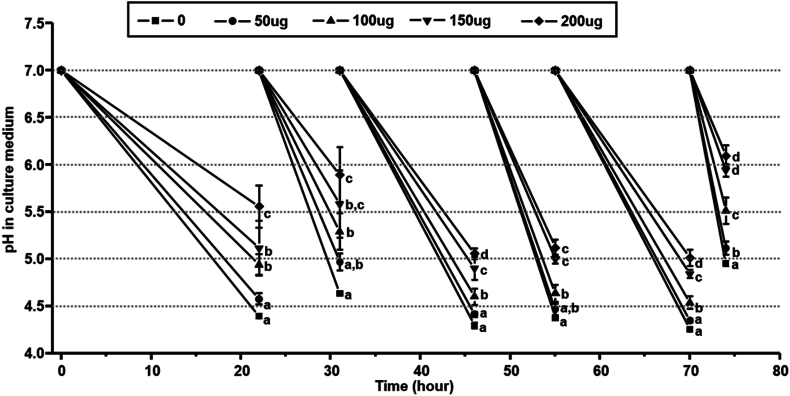
Anti-acidogenic activity of brief usnic acid exposure. pH of the old culture medium after usnic acid brief exposure during the *S. mutans* biofilm development. Data represent mean ± standard deviation of three individual experiments. Values followed by the same superscripts are not significantly different from each other.

Brief exposure of usnic acid strongly diminished the biomass of the *S. mutans* biofilm. As shown in Fig. 3a, brief exposure of usnic acid reduced the biomass by fold of 38–73 % compared to control. Whereas 50 μg/ml of usnic acid diminished the 38 % and 200 μg/ml of usnic acid diminished 73 % of biomass without significantly affecting the viability of bacterial cells in the biofilm matrix (Fig. 3a and b). Brief exposure of usnic acid also inhibited the water-insoluble and iodophilic polysaccharides on *S. mutans* biofilm in concentration dependent manner. As shown in Fig. 3c and d, ≥100 μg/ml of usnic acid significantly reduces the water-insoluble polysaccharide on biofilm whereas, ≥150 μg/ml of usnic acid significantly reduces the iodophilic polysaccharides on *S. mutans* biofilm after twice daily brief exposure. Moreover, ≥100 μg/ml of usnic acid exposure also inhibited the acid production by *S. mutans* biofilm cells in culture medium (Fig. 4).

### 3.4 Microscopic analysis of brief usnic acid exposed biofilm

**Fig. 5:**
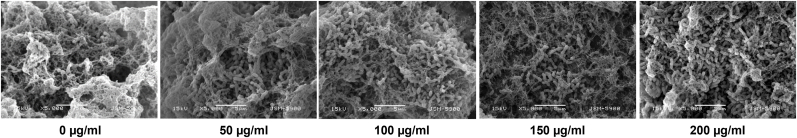
SEM of usnic acid briefly exposed *S. mutans* biofilm. SEM images *S. mutans* biofilm after the twice daily brief exposure with various concentration of usnic acid.

**Fig. 6:**
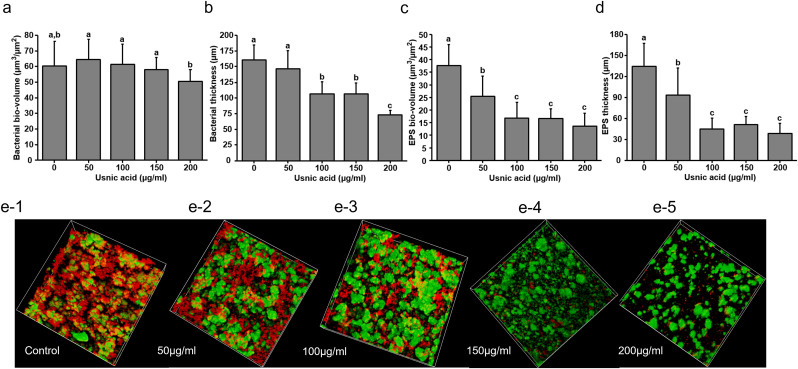
Effect of usnic acid exposure on architecture of *S. mutans* biofilm. Structural architecture of *S. mutans* biofilm after twice daily brief exposure with various concentration of usnic acid. Twice daily usnic acid exposed biofilms were examined by laser scanning microscopy, Red: EPS, Green: bacteria. Quantitative analysis of biovolume and thickness of EPS and bacteria on biofilm were carried out by COMSTAT analysis. (a) Bacterial biovolume, (b) bacterial thickness, (c) EPS biovolume and (d) EPS thickness. e−1, e−2, e−3, e−4 and e−5 are the representative 3D architecture of biofilm briefly exposed with vehicle control, 50 μg/ml, 100 μg/ml, 150 μg/ml and 200 μg/ml of usnic acid respectively. Data represent mean ± standard deviation of three individual experiments. Values followed by the same superscripts are not significantly different from each other.

In order to confirm our biochemical analysis, control and biofilms that are briefly exposed to usnic acid were analyzed by scanning electron microscopy and confocal laser scanning microscopy. SEM images showed the dose dependent decrease of EPS matrix in usnic acid exposed biofilms in compared to control (Fig. 5). Confocal microscopic results revealed that all tested concentrations of usnic acid inhibited the EPS production in *S. mutans* biofilm. As shown in Fig. 6a and b, 200 μg/ml of usnic acid significantly reduces bacterial bio-volume and ≥100 μg/ml significantly disrupted the bacterial thickness of the *S. mutans* biofilm after brief exposure. EPS bio-volume and thickness were significantly reduced by ≥ 50 μg/ml of usnic acid. Whereas 100 μg/ml of usnic acid showed maximum effect in reducing both EPS bio-volume and thickness as similar to 150 and 200 μg/ml (Fig. 6c and d). Confocal microscopy images further confirmed the reduced EPS matrix and disrupted biofilm structure in twice daily usnic acid exposed biofilms in compared to vehicle control (Fig. 6g–h).

### 3.5 Effect brief usnic acid exposure on GTF production by *S. mutans* biofilm

**Fig. 7:**
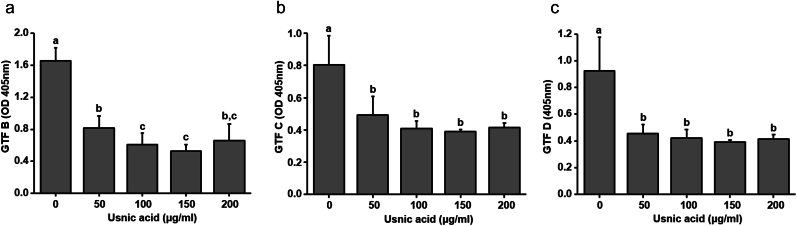
Influence of usnic acid exposure on GTF production. Effect of usnic acid brief exposure on GTFs production by *S. mutans* biofilm cells were evaluated by ELISA. (a) Influence of usnic acid brief exposure on GTF B, (b) GTF C and (c) GTF D production by *S. mutans* biofilm cells. Data represent mean ± standard deviation of three individual experiments. Values followed by the same superscripts are not significantly different from each other.

Brief exposure of usnic acid significantly reduced the GTF production by *S. mutans* cells in biofilm. As shown in Fig. 7, twice daily brief exposure of usnic acid inhibited 51–68 % of GTFB, 39–51 % of GTFC and 52–56 % of GTFD production by *S. mutans* biofilm cells. However, there was no concentration dependent increase in the inhibition of GTF production, ≥100 μg/ml of usnic acid showed about 50 % of inhibition in all three types of GTF production by biofilm cells.

## 4 Discussion

Topical application of antimicrobial agents is considered as a major strategy to control dental diseases like dental caries. It is evident that the concentration of topically applied agents decreases rapidly from oral cavity due to the swallowing and expectoration. This suggests that the agents applied must be able to show their inhibitory effect on a short period of exposure time [[28], [29], [30]]. Considering this fact, this study demonstrated the effect of twice daily topical application of usnic acid on cariogenic properties and maturation of *S. mutans* biofilm.

Although usnic acid has been tested against oral pathogens aiming to prevent dental caries, its inhibitory effect against oral biofilm and cariogenic properties are not yet fully understood. Herein, this study begins with evaluating the effect of usnic acid on acid production and acid tolerance by mature biofilm cells. As shown in Fig. 1, all the concentration of usnic acid significantly disrupted the initial rate of H^+^ ion production and accumulation of H^+^ ion in the period of 2 h. Furthermore, usnic acid also inhibited the ATPase activity of mature biofilm cells in concentration dependent manner. Micro-colonies on the dental biofilm embedded by EPS matrix which hinders the buffering activity of saliva to produce highly acidic microenvironment inside the biofilm matrix [31]. Furthermore, ATPase enzyme in cariogenic bacteria extrudes the intracellular hydrogen ion to balance the extra-intracellular concentrations to support the acid tolerance ability of cariogenic bacteria [32,33]. Consistent production of acid and presence of highly acidic ambience around tooth surface leads to demineralization of the enamel. Hence the bioactive agents which suppress acid production and ATPase activity of cariogenic bacteria might have potential to inhibit dental caries.

In parallel dietary sucrose is continuously utilized by cariogenic bacteria to produce EPS matrix within the biofilm. Especially the glucosyltransferase enzyme produced by *S. mutans* catalyzes the dietary sucrose and produces water insoluble glucan leading to development of complex structure and maturation of biofilm [25,34]. The EPS matrix strongly holds the bacterial communities and establishes the highly dens 3D architecture of biofilm. Due to adhesive nature, glucan is tightly associated with the substrate and difficult to remove completely by mechanical disruption [35,36]. Some studies have revealed that the disruption in EPS formation could reduce the mechanical stability and alteration in the structural integration of microcolonies in biofilm matrix [37,38]. These glucans provide structural stability to biofilms and protect them from environmental assaults and antimicrobial exposure [39]. This glucan matrix is unevenly distributed on the biofilm matrix producing the mushroom shape masses on tooth surfaces resulting in the restriction on the diffusion properties [40,41]. Hence, the inhibition in EPS production could play a vital role to reduce the structure and stability of biofilm matrix followed by the inhibition of cariogenic properties. Herein, usnic acid was tested against the GTF activity of *S. mutans*. Although usnic acid did not inhibit the production of water-soluble polysaccharides, GTF enzymatic activity to produce water insoluble polysaccharide was significantly reduced (Fig. 2). Moreover, twice daily exposure of usnic acid during the biofilm formation significantly lowered the biomass, water soluble polysaccharides and iodophilic polysaccharides within the biofilm matrix without affecting bacterial viability (Fig. 3). Topical exposure of usnic acid not only altered the biomass accumulation on the biofilm matrix, but also inhibited the total acid accumulation on the media showing the potential of topical exposure of usnic acid to reduce the acidogenicity of *S. mutans* biofilm cells (Fig. 3, Fig. 4).

To further investigate the effect of topical exposure of usnic acid, biofilms were examined under the scanning electron microscope and confocal laser scanning microscope. As shown in Fig. 5, the highly dens blanket of EPS matrix found in control biofilms was found to decrease with usnic acid treatment in concentration dependent manner. This result is further confirmed by confocal microscopic examination. As shown in Fig. 6, topical exposure of usnic acid during biofilm formation significantly disrupted the EPS biovolume and thickness within *S. mutans* biofilm matrix. The topical exposure of all tested concentrations of usnic acid significantly disrupted the formation of EPS resulting in the reduction of morphological complexity of biofilm matrix. Highly compacted and EPS encased microcolonies were observed on control, whereas scattered and disconnected microcolonies were observed on biofilms exposed to usnic acid. The inhibition in EPS production by usnic acid treatment resulted the drastic changes in the architecture and complexity of microcolonies. Even though, usnic acid partially inhibited GTF enzymatic activity, EPS formation in the biofilm matrix is drastically altered by topical exposure of usnic acid suggesting that inhibitory effect on EPS formation may not be only due to its inhibitory effect on GTF enzymatic activity. Hence, the effect of usnic acid exposure on the production of GTFs by biofilm cells were evaluated. As shown in Fig. 7, topical application of usnic acid significantly inhibited the production of GTFB, GTFC and GTFD enzymes which are responsible for polysaccharides production. These findings suggest that usnic acid exposure significantly inhibits production of GTF enzymes and partly suppresses their enzymatic activity to inhibit the EPS formation on the biofilm matrix (Fig. 2, Fig. 7). Although usnic acid demonstrated significant antibiofilm potential and a strong inhibitory effect against cariogenic properties, concerns regarding its toxicity remain. The FDA has issued a warning about the use of dietary supplements containing usnic acid due to the possibility of liver toxicity. This warning primarily pertains to the systemic oral use of usnic acid. Since this study evaluates the topical exposure to usnic acid (twice daily, 1-min exposure), the extent of toxicity is likely negligible. However, the potential toxicity of brief usnic acid exposure must be thoroughly evaluated before its use in oral care products.

In conclusion, usnic acid reduces the cariogenic properties and architecture of *S. mutans* biofilm by inhibiting the acid production, acid tolerance and mainly disrupting the EPS formation by biofilm cells. These findings suggest that topical application of usnic acid might be useful as a preventive measure to control the dental caries. However, future research should be focused in evaluating the effect of usnic acid topical exposure against multispecies biofilms and its ability to prevent the dental caries using animal model.

## CRediT authorship contribution statement

**Santosh Pandit:** Writing – review & editing, Writing – original draft, Visualization, Methodology, Investigation, Funding acquisition, Formal analysis, Data curation, Conceptualization. **Mi-A Kim:** Writing – review & editing, Methodology, Investigation, Formal analysis, Data curation. **Ji-Eun Jung:** Writing – review & editing, Methodology, Investigation, Formal analysis, Data curation. **Hyeon-Mi Choi:** Writing – review & editing, Methodology, Formal analysis, Data curation. **Jae-Gyu Jeon:** Writing – review & editing, Supervision, Methodology, Investigation, Funding acquisition, Formal analysis, Conceptualization.

## Funding

This research was supported by funding from Vetenskapsrådet (2020–04096) and the 10.13039/501100003725National Research Foundation of Korea grant (NRF) funded by the Korea government [RS-2023-00246619].

## Declaration of competing interest

The authors declare that they have no known competing financial interests or personal relationships that could have appeared to influence the work reported in this paper.

## Data Availability

Data will be made available on request.
